# Long COVID and Biomarker Dysregulation—A Shift Toward Immune Exhaustion?

**DOI:** 10.3390/medicina61060996

**Published:** 2025-05-28

**Authors:** Anne Kallaste, Kalle Kisand, Agnes Aart, Ahto Salumets, Kai Kisand, Pärt Peterson, Margus Lember

**Affiliations:** 1 Department of Internal Medicine, Tartu University Hospital, L. Puusepa 8, 51014 Tartu, Estonia; margus.lember@ut.ee; 2 Department of Internal Medicine, Institute of Clinical Medicine, University of Tartu, L. Puusepa 8, 51014 Tartu, Estonia; kalle.kisand@ut.ee; 3 South-Estonian Hospital, Meegomäe, 65526 Võru County, Estonia; agnes.aart@leh.ee; 4 Faculty of Science and Technology, Institute of Computer Science, University of Tartu, Narva mnt 18, 51009 Tartu, Estonia; ahto.salumets@ut.ee; 5 Molecular Pathology, Institute of Biomedicine and Translational Medicine, University of Tartu, Ravila 19, 50411 Tartu, Estonia; kai.kisand@ut.ee (K.K.); part.peterson@ut.ee (P.P.)

**Keywords:** post-COVID-19, long COVID, SARS-CoV-2, Olink proteomics, inflammation

## Abstract

*Background and Objectives:* SARS-CoV-2 infection can lead to persistent or newly emerging symptoms lasting for months, a condition known as long COVID (LC). The pathophysiology of LC remains poorly understood, with cytokine dysregulation proposed as a key mechanism, although findings across the studies have been inconsistent. *Materials and Methods:* We conducted a longitudinal study using the Olink^®^ Target 96 Inflammation Panel to assess cytokines in COVID-19 (COV) patients at three months and six months post-infection. These profiles were compared with those of individuals recovering from other upper respiratory tract infections (non-COV). Additionally, we analyzed differences between individuals with LC and those who recovered from COVID-19. Predictive models for LC at three months and sixth months post-infection were developed using inflammatory markers and relevant clinical cofactors, including gender, age, BMI, hemogram, Β2-microglobulin, D-dimers, LDH, AST, ALT, Ferritin, vitamin D, CRP, and the severity of acute COVID-19 infection as classified by WHO criteria. *Results:* We observed a general decline in inflammatory biomarkers in post-COVID-19 patients over time, with only a few cytokines elevated (CCL4 at month 3 and CST5 at month 6) compared to non-COV controls. In LC patients, an early phase of low-grade inflammation transitioned into significant reduction in proinflammatory biomarkers compared to recovered individuals. Rather than indicating immune normalization, this pattern suggests a possible suppression or exhaustion of the immune response in the months following acute infection. Importantly, our predictive modeling demonstrated that this specific cytokine signature, in combination with acute disease severity and clinical cofactors, described well the presence of LC. *Conclusions:* Our findings suggest that inflammation-related biomarker dysregulation following acute SARS-CoV-2 infection evolves dynamically over a six-month period. By the sixth month, compared to the third month, the presence of LC is more accurately predicted by a combination of persistent biomarker alteration and the severity of the initial infection, as defined by WHO criteria. This represents a novel insight, as previous studies have primarily associated LC with elevated proinflammatory markers, whereas our results suggest that immune suppression or exhaustion may play a more prominent role in the later stages.

## 1. Introduction

After the acute phase of COVID-19 (COV), a subset of individuals develops persistent symptoms that can last for months. This condition, known as long COVID (LC), is defined by the WHO as the persistence or emergence of new symptoms three months after the initial SARS-CoV-2 infection, with these symptoms continuing for at least two months without any other explanation [1]. It is estimated that at least 10% of people experience LC after the acute phase of the disease, and although LC is more prevalent among those with severe disease courses, the overall burden is higher among people who have a mild disease course [2].

The clinical sequela of LC is diverse, and over 200 symptoms have been described. According to a meta-analysis, the most common symptoms are mental health symptoms (21.2%); cardiopulmonary symptoms (14.9%); general symptoms like fatigue, myalgia, and fever (14.3%); and neurological symptoms (13.9%) [3]. The pathophysiology behind this heterogeneous clinical picture is complex and not yet thoroughly understood. Multiple risk factors have been suggested to trigger one or several biological mechanisms contributing to the diverse clinical phenotypes of LC. These biological mechanisms may occur at the same time or sequentially over time [4], and extensive research has proposed dysregulated immune response, autoimmunity, reactivation of latent viruses, altered gut microbiota, a persistent SARS-CoV-2 viral reservoir, end-organ damage, and microclots as possible mechanisms [2,4,5,6,7,8].

According to the WHO guidelines [1], the current diagnosis of LC relies upon clinical criteria, causing challenges for early detection and contributing to an increased demand for healthcare resources. Hence, there is an urgent need for diagnostic and prognostic biomarkers to facilitate the prompt diagnosis and clinical management of LC. Although various pathophysiological mechanisms have been suggested, they all involve the inflammatory process. Therefore, proinflammatory biomarkers have been investigated for their potential to diagnose or predict LC. So far, increased levels of proinflammatory cytokines (IFN-β, IFN-λ1, IL-6, TNF-α, and CXCL10) have been demonstrated, although the time periods have varied between studies [9,10,11]. On the contrary, significant reductions of proinflammatory cytokine levels in LC patients have also been demonstrated [12]. Accordingly, these conflicting results need further investigation, especially in longitudinal studies.

The aim of this study was to perform a longitudinal analysis to identify proteomics-based inflammatory biomarkers to characterize and predict LC for up to six months following acute SARS-CoV-2 infection. In addition, the study compared biomarker profiles among individuals who developed LC, those who fully recovered from COVID-19, and patients with other upper respiratory tract infections in order to isolate COVID-specific immune effects.

## 2. Materials and Methods

### 2.1. Study Design and Subjects

We conducted a prospective cohort study that included 60 symptomatic COV patients (positive SARS-CoV-2 RT-RNA from nasal swabs) with different disease severity. The patients were divided into four severity groups (mild, moderate, severe, and critical) based on WHO guidelines as described in our previous study [13].

All consecutive patients with SARS-CoV-2 infection who were hospitalized in Tartu University Hospital from March to May 2020 or turned to the Southern Estonian Hospital emergency medicine department from March to May 2020 were asked to participate. We excluded children and patients with cognitive impairment.

In order to understand possible differences in post-COVID-19 inflammation-related markers compared to other respiratory diseases, we enrolled a control group (named non-COV) consisting of 45 outpatients with upper respiratory tract infections (negative SARS-CoV-2 RT-RNA from nasal swab) who turned to the Southern Estonian Hospital emergency medicine department in spring 2020. The etiology of their upper respiratory infection was unknown, since only a SARS-CoV-2 RT-RNA test using a nasal swab was performed. The non-COV group was age- and sex-matched for the COV patients who were included.

Demographic and clinical data of all the subjects were obtained from the electronic medical records.

COV patients were invited to follow-up visits at month three and six after the acute stage of the disease. All COV patients, except one who only attended the three-month follow-up visit, were present at the six-month follow-up visit. The control group was examined once—at month three. During the follow-up visits, subjects provided blood samples collected into EDTA-vacutainers.

### 2.2. The Follow-Up at Three and Six Months After Acute COV Infection

At follow-up visits, all new symptoms that the patients experienced after the SARS-CoV-2 infection and which were still present at the visit were registered by a physician. Symptoms were divided into seven subgroups for further analysis: (1) general—exercise intolerance, fatigue; (2) neuropsychiatric—anosmia, dysgeusia, memory impairment, tremor, anxiety, paresthesia, hearing impairment, visual impairment; (3) cardiovascular—chest pain, heart failure (new diagnosis/decompensation), hypertension (new diagnosis/decompensation), heart palpitations; (4) respiratory—persistent cough, breathlessness; (5) skeletomuscular—myasthenia, arthralgia; (6) dermatological—hair loss, rash; (7) other symptoms. We also acquired information based on a questionnaire about whether patients had an upper respiratory infection within the previous month before the follow-up visit.

At the third-month follow-up visit of the control group, we ruled out recent acute SARS-CoV-2 infection (negative SARS-CoV-2 RT-RNA from nasal swabs) and previous infection (negative SARS-CoV-2 IgG antibodies in serum).

### 2.3. Profiling of Plasma Inflammation-Related Biomarkers by Olink^®^ Extension Proximity Assay

Blood samples were centrifuged at 1000× *g* and 20 °C for 10 min not more than one hour after collection by venepuncture to minimize the release of inflammation-related biomarkers from blood cells. EDTA plasma samples were stored at −80 °C until testing and thawed only once before measurement.

We used Olink^®^ Target 96 Inflammation Panel (Uppsala, Sweden) to measure 92 inflammation-related biomarkers from EDTA plasma. The method uses two oligonucleotide-conjugated antibodies that bind to the protein epitopes and analysis by a quantitative real-time PCR (RT-PCR) reaction. The raw results were expressed by normalized protein expression (NPX) values on a log2 scale whereby a higher NPX correlates with higher protein expression. Proteins containing NPX values > 50% below the assay’s limit of detection (LOD) were excluded from the analysis. The data were pre-processed by Olink^®^ NPX Manager software (version 2.0.1.175). According to the certificate of the project, intra and inter %CVs are based on control samples (pooled plasma samples) included on each plate. The average intra-assay %CV was 4%, and the average inter-assay %CV was 8% for our project (two plates).

**Figure 1:**
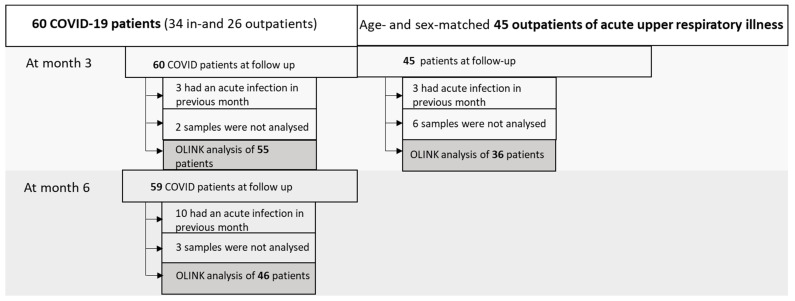
Flow diagram of study subjects at follow-ups at month three and six.

The subjects of both groups (SARS-CoV-2-positive and SARS-CoV-2-negative) who had an acute infection within the previous month before the follow-up visit were excluded from the analysis of inflammatory-related biomarkers. In addition, 11 subjects were not selected for final analysis for technical reasons. Altogether, 55 COV patients at month three, 46 COV patients at month six, and 36 non-COV patients at month three were included in the final analysis of inflammatory biomarkers (Figure 1).

Based on new symptoms after acute SARS-CoV-2 infection, all the COV patients were divided into two groups at months three and six—long COVID (LC) and recovered (R).

To find biomarkers characterizing dysregulated immune response in all post-COVID-19 patients and in LC patients, we compared all COV patients at months three and six with non-COV patients (other respiratory infections) at month three, and LC patients with R patients at months three and six in univariant analysis. To identify biomarkers and clinical features that characterize LC and LC patients with general symptoms (exercise intolerance and fatigue) at months three and six, we used inflammatory-related biomarkers that were significantly associated with univariant analysis and variables according to our clinical experience and the previous literature to perform a multivariant analysis.

The Olink analysis was conducted prior to the availability of vaccination (November 2020).

### 2.4. Data Management and Ethics Approval

Data were collected and managed using REDCap (version 13.4.13; Nashville, TN, USA) electronic data capture tools—hosted at the University of Tartu as previously described [13].

The study was approved by the Ethics Committee for Human Research of the University of Tartu (protocol 318/T-1from 27.05. 2020). All participants provided their written informed consent.

### 2.5. Statistical Analysis

The data were analyzed using SPSS statistics (version 26.0; IBM SPSS Statistics for Windows, Armonk, NY, USA) and R (version 4.2.2; Free Software Foundation, Boston, MA, USA; http://www.r-project.org). All figures were generated using GraphPad Prism (version 9.0.0; GraphPad Software, Boston, MA, USA). Continuous variables with normally distributed data are reported as means and standard deviation (SD), and non-normally distributed data are reported as medians and interquartile range (IQR). Categorical variables are reported as frequency and percentage. For normally distributed data of continuous variables, the independent samples *t*-test was performed, and for non-normally distributed data of continuous variables, the Mann–Whitney *U* or Kruskal–Wallis test was applied. To compare categorical variables, Fisher’s exact test was used.

Logistic regression models (GLMs) implemented in R were applied to investigate associations between biomarker measurements and clinical groups. All GLM analyses, including AUC computation, were performed using the caret package in R. All other statistical analyses (*t*-tests, Mann–Whitney *U* tests, Kruskal–Wallis tests, and Fisher’s exact tests) were conducted using SPSS. All biomarker values were normalized and standardized prior to modeling by centering and scaling based on the mean and standard deviation calculated after applying the Yeo–Johnson transformation. Automated computational pre-filtration was performed by using Caret package in R with training method LOOCV (leave-one-out cross-validation) and modelling with stepAIC (generalized linear model with stepwise bidirectional feature selection). This machine learning pre-filtration method (referred to as LOOCV + stepAIC approach) allows statistical selection of the most significant biomarkers for prognostic modeling. The best model was selected by area under the curve (AUC) estimates. Independent variables in the starting models were selected by previous univariate analysis. Additionally, some routine clinical and laboratory covariates that could affect the analysis were included to improve the prediction of the models. Selected covariates were gender, age, BMI and hemogram (lymphocyte, leucocyte, neutrophils, platelet counts), Β2-microglobulin, D-dimers, LDH, AST, ALT, Ferritin, vitamin D, and CRP.

## 3. Results

### 3.1. Demographics, Clinical Features, and Long-Term Symptoms at Follow-Ups

The COV and non-COV patients were not different in their demographics and comorbidities (Table 1). The mean duration from the symptom onset to the follow-up visits was 97 (±7) days at month three and 189 (±4) days at month six.

**Figure 2:**
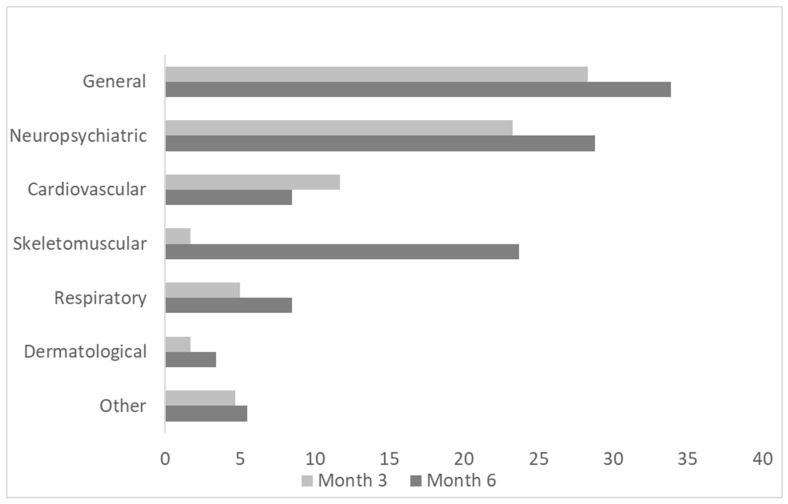
Prevalence of long-term symptoms (%) after acute SARS-CoV-2 infection at month 3 and 6 (general—exercise intolerance, fatigue; neuropsychiatric—anosmia, dysgeusia, memory impairment, tremor, anxiety, paresthesia, hearing impairment, visual impairment; cardiovascular—chest pain, heart failure (new diagnosis/decompensation), hypertension (new diagnosis/decompensation), heart palpitations; respiratory—persistent cough, breathlessness; skeletomuscular—myasthenia, arthralgia; dermatological—hair loss, rash; other).

At months three and six, 48% (*n* = 29) and 66% (*n* = 39) of the COV patients presented at least one new symptom which they did not have before the SARS-CoV-2 infection. The prevalence of the subgroups of symptoms at both follow-ups is provided in Figure 2.

The demographic and clinical data of LC and R patients whose EDTA plasma were used for inflammatory biomarker analysis are provided in Table 2. We found no differences between sex, age, and BMI; however, LC patients had more comorbidities at month six.

At the three-month assessment, more patients in the LC group had required invasive ventilation than in the R group. By the sixth month, the LC group had more patients who had required hospitalization during the acute phase of COVID-19. These patients had been diagnosed with pneumonia or had experienced respiratory failure, necessitating supplementary oxygen.

According to the WHO classification, there were more patients who had had a critical disease course at month three and more patients with a severe disease course at month six in the LC group compared to the R group.

### 3.2. Routine Laboratory Biomarkers at Follow-Ups of Non-COV, LC, and R Patients

As described earlier, subjects with respiratory symptoms within the previous month before follow-ups were excluded from the analysis of inflammation-related biomarkers. As presented in Table 3, we found only one statistically significant difference in routine laboratory biomarkers: at month three, alanine transaminase (ALT) was lower in the non-COV group compared to the LC group, although it remained in the reference range in both groups. There were no differences between R and LC patients.

### 3.3. Univariant Analysis of Inflammatory-Related Biomarkers

#### 3.3.1. Post-Infection Plasma Proteomic Profiles of COV Patients Compared to Non-COV Patients

**Figure 3:**
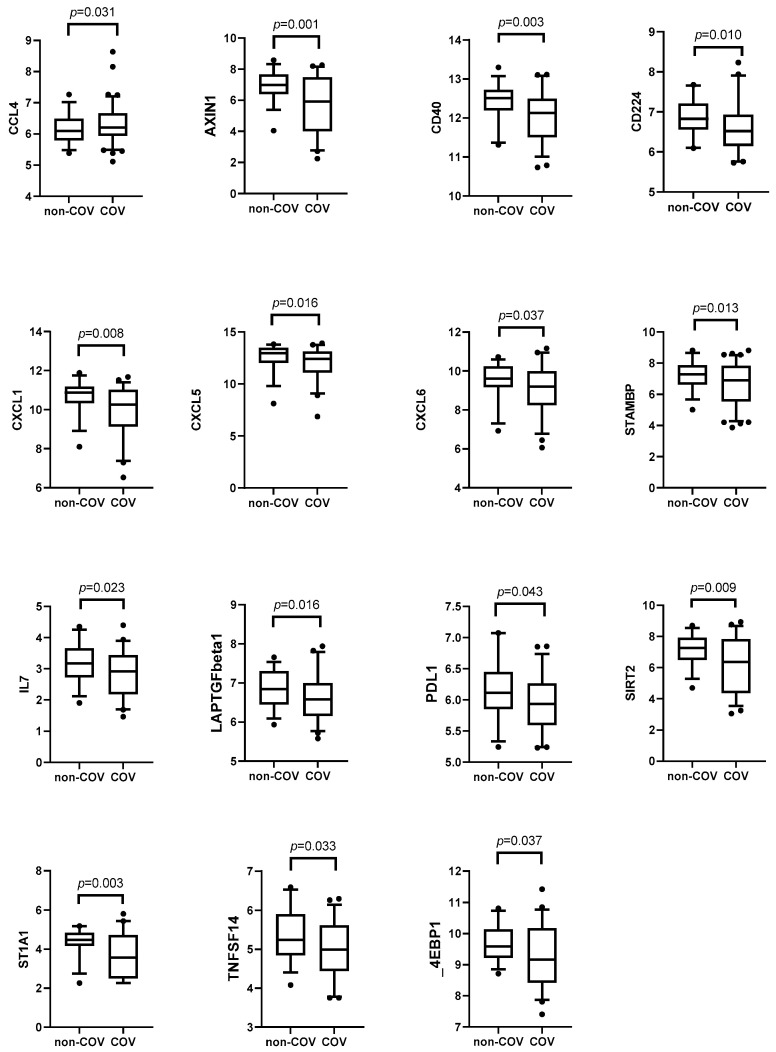
Inflammatory-related biomarkers three months after acute infection in non-COVID (non-COV) and in COVID-19 (COV) patients (Mann–Whitney *U* test). The dots shown outside the boxes indicate individual data points that are unusually high or low compared to the rest of the data.

**Figure 4:**
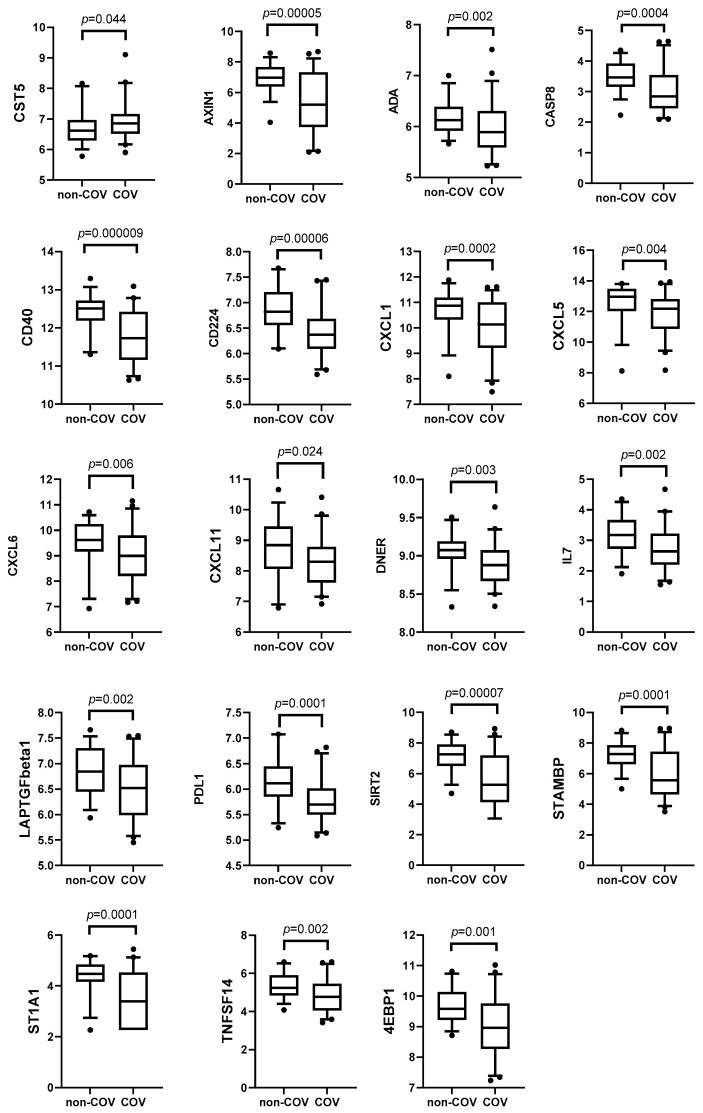
Inflammatory-related biomarkers six months after acute infection in non-COVID (non-COV) and in COVID-19 (COV) patients (Mann–Whitney *U* test). The dots shown outside the boxes indicate individual data points that are unusually high or low compared to the rest of the data.

Altogether, we found 15 plasma proteins of which the levels had changed significantly between COV and non-COV patients three months after the infection (Figure 3). Of these, 14 inflammatory-related biomarkers were decreased in the COV group, and one (CCL4) was increased. Six months after the acute infection, we detected a change in 19 biomarkers, of which 18 were decreased and one (CST5) increased in the COV group compared to the non-COV patients (Figure 4). We found that the signature of altered biomarkers largely overlapped at months 3 and 6, as the decrease in 12 inflammation-related biomarkers was present at both time points (Figure 3 and Figure 4).

#### 3.3.2. Post-Infection Plasma Proteomic Profiles of LC Compared to R Patients

**Figure 5:**
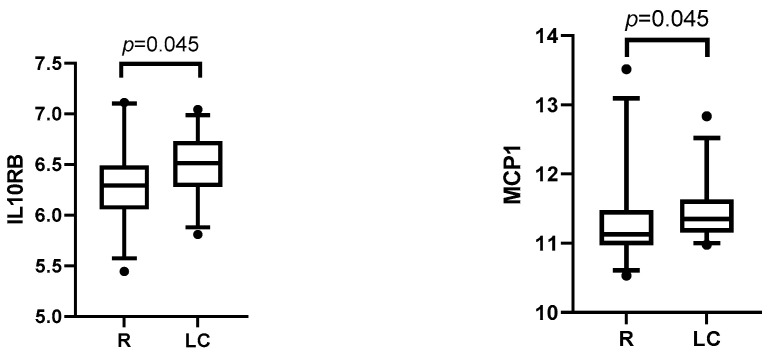
Inflammatory-related biomarkers three months after acute SARS-CoV-2 infection in recovered COVID-19 (R) and long COVID (LC) patients (Mann–Whitney *U* test). The dots shown outside the boxes indicate individual data points that are unusually high or low compared to the rest of the data.

**Figure 6:**
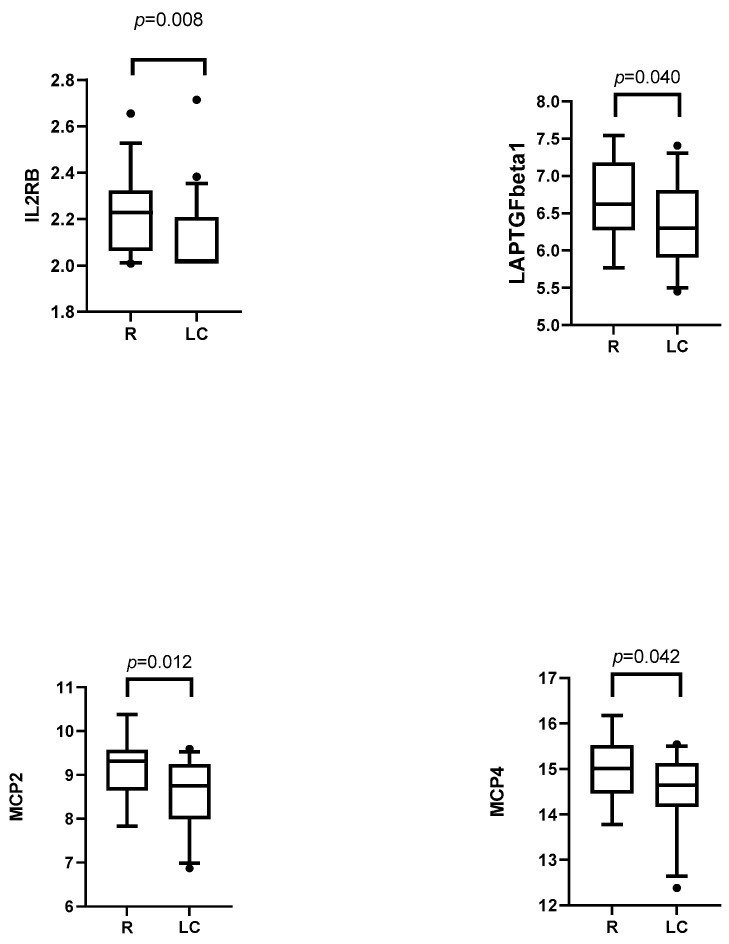
Inflammatory-related biomarkers six months after acute SARS-CoV-2 infection in recovered COVID-19 (R) and long COVID (LC) patients (Mann–Whitney *U* test). The dots shown outside the boxes indicate individual data points that are unusually high or low compared to the rest of the data.

We only found a few LC-specific inflammatory biomarker changes in the third month. Two of these, MCP1 and IL10RB, were increased in LC patients compared to R patients (Figure 5). However, the increase in these biomarkers did not persist at month 6 of the post-infection period, and instead, we saw significantly lower levels in four inflammatory biomarkers (IL2RB, LAPTGFbeta1, MCP2, and MCP4) in LC patients (Figure 6).

### 3.4. Multivariant Analysis of Inflammatory-Related Biomarkers

The GLMs with stepwise bidirectional selection and leave-one-out cross-validation (stepAIC + LOOCV) of BMs were used to compare the following groups: (1) LC vs. R and (2) long COVID with general symptoms (GEN-LC) vs. R. Biomarkers in the starting models were selected from a previous univariate analysis. This approach enabled us to assess the combined effects of multiple cytokines while adjusting for confounding factors. By accounting for individual variability and potential interactions, GLMs improve the robustness of the analysis and help uncover independent associations with LCOV that might be overlooked in univariate models.

The prediction models for LC containing biomarkers alone or with the combination of clinical and laboratory covariates (gender, patient age, BMI, WHO group, 5-diff hemogram, Β_2_-microglobulin, D-dimers, LDH, AST, ALT, Ferritin, vitamin D, and CRP) showed modest performance (AUC 0.3–0.73, Table 4 and Table 5); however, the predictive power was better at month 6 compared to month 3. We found that LC at month 3 was characterized by a statistically significant increase in CD5; however, adding ferritin into the model resulted in the replacement of CD5 with IL10RB (M6, Table 4). Of note, WHO group data (severity of COVID-19 at the acute period) did not associate statistically significantly with LC status at month 3 and, therefore, was not selected in the best model.

At month 6, MCP2 and IL2RB were selected in the final model for LC (model M3, Table 4); however, MCP2 was the only biomarker statistically significantly associated (OR = 0.40 for MCP2, *p* = 0.02). Moreover, the severity of acute illness (assessed by WHO group data during the acute phase) strongly predicted LC at month 6 (OR = 2.42, 95%CI 1.14–15.9, M4 model, Table 5).

The final models for GEN-LC (combining exercise intolerance and fatigue) at month three listed six biomarkers (TNF, CXCL1, TRANCE, TNFRSF9, IL10RB, SLAMF1); however, only TRANCE was statistically significant (OR = 7.87, 95%CI 1.21–51.1, *p* = 0.031), and the model’s performance was relatively poor (AUC = 0.60 for model M7; Table 4) due to relatively low specificity (33%) of the prediction. Of note, including WHO group data in the month 3 model did not improve the prediction of GEN-LC.

At month six, three inflammation-related biomarkers (MCP2, TRAIL, and uPA) were selected for the final model for general symptoms; however, the model performance was weak (AUC = 0.59). Of note, including WHO group data in the model notably improved the prediction (AUC = 0.83, M6, Table 5). Summarizing the results, we demonstrated that general symptoms of LC at month six were associated statistically significantly with an increase in uPA (plasminogen activator) and with more severe COV in the acute phase of the infection.

## 4. Discussion

Here, we studied the changes of 92 inflammatory markers in COV compared to other respiratory infections and the longitudinal biomarker differences in LC and R patients three and six months after the acute infection.

Since the beginning of the pandemic, there has been considerable debate surrounding the distinctive nature of COVID-19’s disease course compared to other respiratory infections, notably influenza. Despite observed similarities, a recent study has disclosed that COV imposes a greater burden of acute and post-acute health loss than seasonal influenza, thereby affirming that SARS-CoV-2 is beyond the classification of a typical respiratory viral infection [14]. Moreover, in contrast to influenza, SARS-CoV-2 manifests a substantially higher risk for multiorgan involvement [14]. We found only a few biomarkers that were elevated (CCL4 at month three and CST5 at month six) in COV compared to non-COV patients in follow-up. Interestingly, most of the biomarkers with significant alterations (STAMBP, CXCL1, CXCL6, CXCL5, SIRT2, AXIN1, CD40, ST1A1, IL7, CD224, LAPTGFbeta1, TNFSF14, PDL1, 4E-BP1) were decreased at the third month, with a further decline at the sixth month compared to controls. This finding emphasizes the magnitude of immune dysregulation in post-COV patients compared to other respiratory tract infections, associated with prior systemic multiorgan SARS-CoV-2 infection. A recent review elucidated parallels and disparities in cytokine profiles during the acute phase of COV and influenza [15], but there have not been many comparable studies, especially in the post-acute period. Even though the etiology of respiratory infections in our control group remains unknown, and the course of the disease was mild compared to COV patients, our findings add valuable insights into discerning the immune profiles between post-COVID-19 status and convalescent stages of other respiratory viral infections.

Although over two hundred putative biomarkers have been proposed for LC [8], a comprehensive review underscored the low predictive power of routine laboratory markers for LC, including CRP, neutrophil/lymphocyte ratio, LDH, and others. In agreement, we did not find routine measurements to differentiate between LC and R subgroups. [16]. We therefore concur that using typical laboratory indications alone is insufficient to ascertain a person’s LC status.

In our analysis of 92 inflammatory biomarkers, we identified a subset of plasma proteins which were differently expressed in LC and R patients. Interestingly, we saw a considerable time-dependent realignment of the inflammatory protein levels between the time points. At month three, LC patients had elevated IL10RB and MCP-1, whereas by month six, levels of several proteins (monocyte chemoattractant proteins MCP2/CCL8 and MCP4/CCL13, lymphopenia associated IL2RB, and TGF-signaling LAP/TGFb1) had fallen. The importance of these findings needs further clarification.

IL-10RB is a cell surface receptor activated by IL-10 and other class 2 cytokines [17]. Elevated levels of IL-10 were found in severe COV, which may be explained by non-classical proinflammatory actions of IL-10 or its resistance to anti-inflammatory signaling [18]. MCP-1 is overexpressed during the acute phase of SARS-CoV-2 infection and has been linked to the severity of the disease [19,20]. We found that MCP-1 was elevated at month three, which could be connected to the prolonged inflammatory response that occurs during the acute phase [21] (also known as a “cytokine storm”) [22]. Interestingly, new data with a longer follow-up period (up to 12 months) have demonstrated elevated MCP-1 in LC, indicating a longer low-lever inflammation in certain LC endotypes [23]. MCP-2, MCP-4, IL2RB, and LAP-TGF-β1 were decreased at six months. These proteins have been linked to a severe course of COVID-19; for example, MCP-2 and MCP-4 are chemokines that recruit immune cells to the infection site. MCP-2 has been demonstrated to be elevated in severe COV cases during the acute phase [24]. MCP-4 has been poorly studied in COV patients, yet it has multiple proinflammatory properties [25]. IL2RB is a receptor subunit via which IL-2 promotes T-cell proliferation and differentiation, particularly the survival and function of regulatory T-lymphocytes [26]. A meta-analysis showed increased levels of IL-2 in COV patients during the acute phase, compared to controls, but did not show differences in disease severity [27]. One study showed a positive association of IL2R with severe COV [21]. Notably, LAP-TGF-β1, a latent form of TGF-β1 activated during tissue injury and inflammation, is linked to microvascular injury in severe COV and subsequent fibrosis development [28]. Dysregulation of IL-2 and TGF-β1 has been documented in other long COV studies, with conflicting findings regarding IL-2 levels [12,29] and elevated TGF-β levels in LC patients with pulmonary fibrosis [30].

Our study supports the concept that LC individuals undergo sustained low-level inflammation that may be related to several factors—SARS-CoV-2 persistence, compromised gut integrity, and reactivation of latent viruses [2,4,5,6,7,8]. Interestingly, we found that increased levels of proinflammatory biomarkers in LC are replaced with decreased levels of inflammatory biomarkers at six months. We propose, as suggested in earlier publications [12], that LC may be partly linked to post-infection exhaustion of innate immune mechanisms—a phenomenon termed post-infectious immune paralysis in severe cases [31].

Also, the observed decline in IL2RB at month six may contribute to the autoimmune processes in LC pathogenesis, given the crucial role of regulatory T-lymphocytes in maintaining self-tolerance; blocking IL-2 signaling has also been linked to autoimmune responses [26].

Modeling of post-acute inflammation-related biomarker profiles following acute COV infection supported our initial findings of sustained realignment of the immune system for at least six months. Improvement of the models over time may indicate the reconfiguration in inflammatory biomarker profiles as the dynamic nature of LC and gradual exacerbation of initial symptoms occurs after half a year of follow-up, as has been described in a recent study [32]. This may suggest that the continual realignment of inflammation-related biomarker profiles may play a more significant role in the manifestation and aggravation of LC symptoms over time.

Prior research has highlighted the significance of dividing LC into subgroups to identify the underlying pathophysiology. We found the models to perform better in patients who had general symptoms like exercise intolerance and fatigue. In this subgroup, we noticed an increase in urokinase plasminogen activator (uPA) at month six, which was not detectable in a cohort of all LC patients. uPA converts plasminogen into plasmin, which is essential for controlling fibrinolysis [33]. While hypercoagulation and platelet hyperactivation are well documented in acute SARS-CoV-2 infection, studies also reveal persistent clotting abnormalities in LC patients. For instance, plasma from LC patients contains amyloid accumulations (microclots) resistant to fibrinolysis [34]. These amyloid deposits have been identified in the muscles of LC patients experiencing fatigue and post-exertional malaise [35]. Our observation of elevated uPA expression points to a clotting abnormality in a subset of patients with exercise intolerance and fatigue.

Our study has several strengths. To mitigate potential confounding variables, we excluded patients with recent upper respiratory tract infections. Also, we compared inflammation-related biomarkers in the samples collected at the same timepoint. Furthermore, we included a control group with other upper respiratory infections. Our research’s strength lies in creating predictive models for long COVID that are based on immunological factors and other covariates that are clinically relevant. In order to decrease over-fitting in our statistical models, we also employed the LOOCV method and a machine learning strategy.

It is, however, important to recognize some of our study’s shortcomings. Our control group of respiratory tract infections (non-COV) did not include hospitalized and severe cases. Additionally, blood samples from patients in the non-COV group were acquired just once, at the three-month mark following infection. Given the higher prevalence of severe disease that leads to LC formation, our findings on plasma proteomic profiles may not be generally applicable to patients with milder disease histories who have long-lasting symptoms. Furthermore, we did not carry out a thorough examination of immune cell populations.

In conclusion, our findings with different inflammatory-related biomarkers suggest that the course of LC is a dynamic process of immune dysfunction including the interplay of different inflammation-related biomarkers that may play a role in development of LC symptoms. Although numerous inflammatory-related markers alone could not predict the presence of LC indicating the complex nature of LC, our study still provides valuable insight emphasizing the role of inflammation, immune dysregulation, and altered coagulation in LC patients. Furthermore, the refining of our prediction models over time implies that the definition of LC becomes clearer by month six, emphasizing the importance of long-term monitoring.

## Figures and Tables

**Table 1:** Demographics and clinical characteristics of COV patients and non-COV patients.

	COV (*n* = 60)	Non-COV (*n* = 45)	*p*-Value *
Age, mean (SD) (years)	56.9 (16.4)	53.8 (13.9)	0.403
Male gender, n (%)	28 (46.7)	22 (48.9)	0.845
BMI, mean (SD)	28.9 (4.3)	29.6 (6.3)	0.95
Chronic diseases and comorbidities			
Any comorbidity, n (%)	37 (61)	32 (71)	0.407
Hypertension, n (%)	21 (35)	16 (36)	1.0
COPD, n (%)	2 (3.3)	1 (2.2)	1.0
Asthma, n (%)	3 (5)	2 (4.4)	1.0
Diabetes, n (%)	5 (8.3)	3 (6.7)	1.0
Coronary artery disease, n (%)	7 (11.7)	3 (6.7)	0.510
Cerebrovascular disease, n (%)	2 (3.3)	1 (2.2)	1.0
Tumor, n (%)	1 (1.6)	4 (8.8)	0.389

* *p*-values were calculated for normally distributed continuous data with an independent samples *t*-test, for non-normally distributed continuous data with the Mann–Whitney *U* test, and with Fisher’s exact test for categorical variables. The significance level is *p*-value < 0.05. BMI, body mass index; COPD, chronic obstructive pulmonary disease; COV, COVID-19; non-COV, non-COVID-19; SD, standard deviation.

**Table 2:** Demographic and clinical characteristics of R * and LC * groups at follow-up visits.

	At Month 3			At Month 6		
	R (*n* = 28)	LC (*n* = 27)	*p*-Value **	R (*n* = 18)	LC (*n* = 28)	*p*-Value **
Age, mean (SD) (years)	56 (17)	56 (15)	0.933	59 (17)	62 (16)	0.547
Male gender, n (%)	13 (46)	17 (63)	0.282	9 (50)	14 (50)	1.000
BMI, mean (SD)	28.1 (4.6)	30.1 (4.1)	0.069	28.3 (4.8)	29.8 (4.5)	0.291
Chronic diseases and comorbidities						
Any comorbidity, n (%)	17 (60.7)	16 (59.3)	1.000	9 (50)	22 (78.6)	0.046
Hypertension, n (%)	9 (32.1)	8 (29.6)	1.000	5 (27.8)	16 (57.1)	0.072
COPD, n (%)	1 (3.6)	1 (3.7)	1.000	0	2 (7.1)	0.513
Asthma, n (%)	2 (7.1)	1 (3.7)	1.000	1 (5.6)	2 (7.1)	1.000
Diabetes, n (%)	2 (7.1)	3 (11.1)	0.669	0	5 (17.9)	0.140
Coronary artery disease, n (%)	4 (14.3)	2 (7.4)	0.669	2 (11.1)	5 (17.9)	0.688
Cerebrovascular disease, n (%)	2 (7.1)	0	0.491	0	2 (7.1)	0.513
Tumor, n (%)	0	4 (14.8)	0.051	1 (5.6)	3 (10.7)	1.000
Treatment in acute phase						
Hospitalized, n (%)	11 (39.3)	16 (59.3)	0.181	6 (33.3)	19 (67.9)	0.034
Parenteral antibiotics, n (%)	6 (21.4)	12 (44.4)	0.089	3 (16.7)	13 (46.4)	0.058
Hydroxychloroquine, n (%)	9 (32.1)	13 (48.1)	0.277	3 (16.7)	17 (60.7)	0.006
Glucocorticoids, n (%)	0	1 (3.7)	0.491	0	1 (3.6)	1.000
Supplemental oxygen, n (%)	10 (35.7)	16 (59.3)	0.108	5 (27.8)	20 (71.4)	0.006
NIV, n (%)	1 (3.6)	6 (22.2)	0.051	0	5 (17.9)	0.140
Invasive ventilation, n (%)	0	6 (22.2)	0.010	0	3 (10.7)	0.270
ICU admission, n (%)	2(7.1)	7 (25.9)	0.078	1 (5)	5 (17.9)	0.380
WHO group, n (%):						
Mild	8 (28.6)	5 (18.6)	0.528	7 (38.9)	4 (14.3)	0.08
Moderate	11 (39.3)	6 (22.2)	0.245	7 (38.9)	4 (14.3)	0.08
Severe	9 (32.1)	9 (33.3)	1.000	4 (22.2)	16 (57.1)	0.032
Critical	0	7 (25.9)	0.004	0	4 (14.3)	0.144
Acute complications						
Pneumonia, n (%)	10 (35.7)	16 (59.3)	0.108	5 (27.8)	20 (71.4)	0.006
Respiratory failure, n (%)	8 (28.6)	13 (48.1)	0.112	4 (22.2)	16 (57.1)	0.032

* Only patients with further inflammation-related biomarker analysis (OLINK) were included; ** *p*-values were calculated for normally distributed continuous data with an independent samples *t*-test, non-normally distributed continuous data were calculated with a Mann–Whitney *U* test, and Fisher’s exact test was used for categorical variables. The significance level is *p*-value < 0.05. BMI, body mass index; COPD, chronic obstructive pulmonary disease; LC, long COVID; R, recovered COVID-19 patients; ICU, intensive care unit; NIV, non-invasive ventilation; SD, standard deviation. The grey background in the *p*-values is used to indicate significant results (*p* < 0.05).

**Table 3:** Routine laboratory biomarkers of non-COV *, R *, and LC * patients at month three and six.

	At Month 3				At Month 6			
Laboratory Biomarkers, Median (IQR)	Non-COV (*n* = 36)	R (*n* = 28)	LC (*n* = 27)	*p*-Value **	Non-COV *** (*n* = 36)	R (*n* = 18)	LC (*n* = 28)	*p*-Value **
Leukocytes (E9/L)	5.8 (4.9–7.0)	5.7 (5.0–6.7)	6.4 (4.8–7.0)	0.482	5.8 (4.9–7.0)	6.2 (5.4–6.9)	6.0 (5.3–7.4)	0.59
Lymphocytes (E9/L)	1.9 (1.6–2.1)	1.9 (1.7–2.3)	2.0 (1.6–2.6)	0.640	1.9 (1.6–2.1)	2.2 (1.5–2.6)	2.1 (1.7–2.6)	0.763
Neutrophils (E9/L)	3.2 (2.5–3.8)	3.0 (2.6–3.8)	3.2 (2.4–3.8)	0.869	3.2 (2.5–3.8)	3.4 (2.7–4.0)	3.3 (3.0–4.2)	0.763
Thrombocytes (E9/L)	217 (190–238)	236 (208–278)	242 (216–281)	0.094	217 (190–238)	250 (208–278)	228 (204–261)	0.131
CRP (mg/L)	1.5 (1–3.75)	1.0 (1.0–3.0)	1.0 (1.0–2.0)	0.433	1.5 (1–3.75)	2.0 (1.0–3.0)	1.0 (1.0–2.8)	0.128
ALT (U/L)	17.5 (12–23.75)	23 (15–32)	26 (19–34)	0.028 ****	17.5 (12–23.75)	25 (17–38)	21 (16–27)	0.114
Ferritin (µg/L)	160 (75–245)	140 (95–220)	92 (56–234)	0.302	160 (75–245)	161 (70–204)	129 (74–181)	0.365
NT-proBNP (ng/L)	62 (37–112)	64 (44–102)	67 (36–99)	0.663	62 (37–112)	66 (34–150)	65 (35–147)	0.763
Creatinine (µmol/L)	70 (66–80)	69 (57–84)	71 (55–86)	0.702	70 (66–80)	72 (62–81)	67 (58–80)	0.763
Bilirubin (µmol/L)	8.7 (7.0–11.7)	7.4 (6.0–9.9)	8.3 (5.9–11.2)	0.597	8.7 (7.0–11.7)	7.4 (5.5–10.0)	8.2 (7.0–12.0)	0.763
D-dimers (mg/L)	0.31 (0.27–0.51)	0.32 (0.27–0.63)	0.32 (0.27–0.55)	0.976	0.31 (0.27–0.51)	0.37 (0.32–0.54)	0.39 (0.29–0.56)	0.663

* Only patients with further inflammation-related biomarker analysis (OLINK) were included; ** *p*-values were calculated for normally distributed continuous data with a one-way ANOVA test and, for non-normally distributed continuous data, with the Kruskal–Wallis test. Statistically significant pairwise comparison is adjusted for three hypotheses. The significance level is *p*-value < 0.05; *** biomarkers at follow-up month 3; **** further pairwise comparisons demonstrated statistically significant difference between non-COV and LC (*p* = 0.031); ALT, alanine aminotransferase; CRP, C-reactive protein; IQR, interquartile range; LC, long COVID; non-COV, non-COVID; R, recovered COVID-19 patients. The grey background in the *p*-values is used to indicate significant results (*p* < 0.05).

**Table 4:** Prediction models for LC and GEN-LC at three months after acute infection.

Comparable Subgroups (Group Sizes)	Model *	Independent Variables in the Final Model **	Coefficient Estimate ***	Adjusted OR (CI95%) ****	Adjusted *p*-Value *****	Model AUC (Sensitivity, Specificity)
LC vs. R (27/28)	M1	**CD5**	0.66698	1.95 (1.025; 3.70)	0.042	0.56 (0.64; 0.44)
	M2 ^a^	D-dimers	−18.3908	10−8 (10−21; 13510)	0.196	0.30 (0.30; 0.37)
		AST	−1.5196	0.22 (0.04; 1.1)	0.068	
		Age	−0.7566	0.47 (0.18; 1.23)	0.124	
		Leucocytes	−0.6806	0.51 (0.24; 1.08)	0.080	
		Female gender	−0.5542	0.57 (0.28; 1.2)	0.140	
		ALT	1.0891	2.97 (0.76; 11.7)	0.118	
		IL10RB	0.9672	2.63 (0.89; 7.8)	0.080	
		TNFRSF9	0.8655	2.38 (0.81; 7.0)	0.117	
		IL6	0.7328	2.08 (0.93; 4.67)	0.076	
	**M3** ^a^	Ferritin	−0.61458	0.54 (0.26; 1.13)	0.10	**0.62 (0.59; 0.63)**
		**IL10RB**	0.79028	2.2 (1.07; 4.5)	0.032	
		BMI	0.61753	1.85 (0.98; 3.53)	0.06	
GEN-LC vs. R (15/28)	**M4 ^b^**	TNF	−2.7214	0.066 (0.003; 1.51)	0.090	**0.60 (0.82; 0.33)**
		CXCL1	−0.8787	0.42 (0.15; 1.17)	0.098	
		**TRANCE**	2.0625	7.87 (1.21; 51.1)	0.031	
		TNFRSF9	1.6732	5.33 (0.82; 34.6)	0.080	
		IL10RB	1.4862	4.42 (0.86; 22.6)	0.075	
		SLAMF1	0.9473	2.58 (0.78; 8.57)	0.122	

* The best final model of comparable subgroups in bold. ** Independent variables of the final model after Caret package “glmStepAIC” stepwise selection (by iteratively adding and removing predictors in the predictive model) in order to find the subset of variables in the data set resulting in the best performing model with lowers prediction error; statistically significant biomarkers of the final model are in bold; *** independent variables pre-processed (“centered” and “scaled” in caret) for better comparison of coefficients. **** Models were trained by using LOOCV method (leave-one-out cross-validation) for adjusted OR and *p*-value; ***** non-COVID-19 (control group) = outpatients with acute upper respiratory illnesses but with COVID-19-negative PCR test. Independent variables in the starting models selected by previous univariate analysis: M1: Olink inflammation-related biomarkers (MCP1 + IL10RB + IL6 + CD5 + SLAMF1 + TNFRSF9)*;* M2: M1 adjusted for all selected covariates^a^; M3: M1 adjusted for Ferritin and BMI; M4: Olink inflammation-related biomarkers (IL10RB + CXCL1 + IL6 + MCP1 + CXCL10 + IL15RA + TNF + FGF5 + CSF1 + IL10 + ENRAGE + TNFRSF9 + SLAMF1 + CDCP1 + MCP3 + TRANCE + CD6 + CXCL9 + IFNγ). ^a^ Covariates were selected in our study according to our clinical experience and the previous literature. Selected covariates were gender, age, BMI, and routine lab tests for lymphocyte, leucocyte, neutrophils, platelet counts, B_2_-microglobulin, D-dimers, LDH, AST, ALT, Ferritin, vitamin D, and CRP; ^b^ including WHO group did not improve the model; n—cases in the groups; AUC—area under the receiver operating characteristic (ROC) curve; OR (CI95%)—odds ratio and 95% confidence interval for it; ALT, alanine aminotransferase; AST, aspartate aminotransferase; BMI, body mass index; CRP, C-reactive protein; LC, long COVID; GEN-LC, long COVID with general symptoms; R, recovered COVID-19 patients.

**Table 5:** Prediction models for LC and GEN-LC at six months after acute infection.

Comparable Subgroups (Group Sizes)	Model *	Independent Variables in the Final Model **	Coefficient Estimate ***	Adjusted OR (CI95%) ***	Adjusted *p*-Value ****	Model AUC (Sensitivity, Specificity)
LC vs. R (28/18)	M1	**MCP2**	−0.9241	0.40 (0.18; 0.87)	0.020	0.70 (0.50; 0.71)
		IL2RB	−0.6255	0.53 (0.26; 1.08)	0.081	
	**M2**	MCP2	−0.8227	0.44 (0.18; 1.1)	0.078	**0.73 (0.61; 0.75)**
		**WHO group**	0.8846	2.42 (1.14; 5.1)	0.021	
GEN-LC vs. R (17/18)	M3	**MCP2**	−1.42641	0.24 (0.08; 0.75)	0.0142	0.59 (0.55; 0.59)
		TRAIL	−0.85045	0.43 (0.15; 1.22)	0.1133	
		**uPA**	1.43955	4.22 (1.12; 15.9)	0.0339	
	**M4**	TRAIL	−0.9255	0.40 (0.13; 1.17)	0.095	**0.83 (0.78; 0.88)**
		**WHO group**	2.4988	12.2 (2.35; 63)	0.0029	
		**uPA**	1.3796	3.97 (1.06; 14.9)	0.041	

* The best final model of comparable subgroups in bold; ** independent variables of the final model after caret “glmStepAIC” stepwise selection (by iteratively adding and removing predictors in the predictive model) in order to find the subset of variables in the data set resulting in the best performing model with lowers prediction error; statistically significant biomarkers of the final model are in bold; *** independent variables pre-processed (“centered” and “scaled” in caret) for better comparison of coefficients; **** caret models were trained by using LOOCV method (leave-one-out cross-validation) for adjusted OR and *p*-value. Independent variables in the starting models selected by previous univariate analysis: M1: Olink inflammation-related biomarkers (MCP2 + LAPTGFbeta1 + IL2RB); M2: MCP2 + WHOgroup; M3: Olink inflammation-related biomarkers (uPA + EBP1 + PDL1 + TRAIL + LAPTGFbeta1 + MCP2 + IL2RB); M4: uPA + EBP1 + PDL1 + TRAIL + WHOgroup; ALT, alanine aminotransferase; AST, aspartate aminotransferase; BMI, body mass index; CRP, C-reactive protein; LC, long COVID; GEN-LC, long COVID with general symptoms; R, recovered COVID-19 patients.

## Data Availability

The data described in the manuscript are available upon reasonable request.

## References

[1] World Health Organization (2021). A Clinical Casedefinition of Post COVID-19 Condition by a Delphiconsensus.

[2] Davis H.E., McCorkell L., Vogel J.M., Topol E.J. (2023). Long COVID: Major findings, mechanisms and recommendations. Nat. Rev. Microbiol..

[3] Natarajan A., Shetty A., Delanerolle G., Zeng Y., Zhang Y., Raymont V., Rathod S., Halabi S., Elliot K., Shi J.Q. (2023). A systematic review and meta-analysis of long COVID symptoms. Syst. Rev..

[4] Perumal R., Shunmugam L., Naidoo K., Karim S.S.A., Wilkins D., Garzino-Demo A., Brechot C., Parthasarathy S., Vahlne A., Nikolich J.Ž. (2023). Long COVID: A review and proposed visualization of the complexity of long COVID. Front. Immunol..

[5] Su S., Zhao Y., Zeng N., Liu X., Zheng Y., Sun J., Zhong Y., Wu S., Ni S., Gong Y. (2023). Epidemiology, clinical presentation, pathophysiology, and management of long COVID: An update. Mol. Psychiatry.

[6] Proal A.D., VanElzakker M.B. (2021). Long COVID or Post-acute Sequelae of COVID-19 (PASC): An Overview of Biological Factors That May Contribute to Persistent Symptoms. Front. Microbiol..

[7] Tsilingiris D., Vallianou N.G., Karampela I., Christodoulatos G.S., Papavasileiou G., Petropoulou D., Magkos F., Dalamaga M. (2023). Laboratory Findings and Biomarkers in Long COVID: What Do We Know So Far? Insights into Epidemiology, Pathogenesis, Therapeutic Perspectives and Challenges. Int. J. Mol. Sci..

[8] Lai Y.-J., Liu S.-H., Manachevakul S., Lee T.-A., Kuo C.-T., Bello D. (2023). Biomarkers in long COVID-19: A systematic review. Front. Med..

[9] Peluso M.J., Lu S., Tang A.F., Durstenfeld M.S., Ho H.-E., Goldberg S.A., Forman C.A., Munter S.E., Hoh R., Tai V. (2021). Markers of Immune Activation and Inflammation in Individuals with Postacute Sequelae of Severe Acute Respiratory Syndrome Coronavirus 2 Infection. J. Infect. Dis..

[10] Phetsouphanh C., Darley D.R., Wilson D.B., Howe A., Munier C.M.L., Patel S.K., Juno J.A., Burrell L.M., Kent S.J., Dore G.J. (2022). Immunological dysfunction persists for 8 months following initial mild-to-moderate SARS-CoV-2 infection. Nat. Immunol..

[11] Zhao J., Schank M., Wang L., Dang X., Cao D., Khanal S., Nguyen L.N., Zhang Y., Wu X.Y., Adkins J.L. (2022). Plasma biomarkers for systemic inflammation in COVID-19 survivors. Proteom.–Clin. Appl..

[12] Williams E.S., Martins T.B., Shah K.S., Hill H.R., Coiras M., Spivak A.M., Planelles V. (2022). Cytokine Deficiencies in Patients with Long-COVID. J. Clin. Cell. Immunol..

[13] Kallaste A., Kisand K., Aart A., Peterson P., Lember M. (2021). Antibody levels remain high to one-year’s follow-up after moderate and severe COVID-19, but not after mild cases. Infect. Dis..

[14] Xie Y., Choi T., Al-Aly Z. (2023). Long-term outcomes following hospital admission for COVID-19 versus seasonal influenza: A cohort study. Lancet Infect. Dis..

[15] Pacheco-Hernández L.M., Ramírez-Noyola J.A., Gómez-García I.A., Ignacio-Cortés S., Zúñiga J., Choreño-Parra J.A. (2022). Comparing the Cytokine Storms of COVID-19 and Pandemic Influenza. J. Interf. Cytokine Res..

[16] Espín E., Yang C., Shannon C.P., Assadian S., He D., Tebbutt S.J. (2023). Cellular and molecular biomarkers of long COVID: A scoping review. eBioMedicine.

[17] Ahn D., Prince A. (2020). Participation of the IL-10RB Related Cytokines, IL-22 and IFN-λ in Defense of the Airway Mucosal Barrier. Front. Cell. Infect. Microbiol..

[18] Islam H., Chamberlain T.C., Mui A.L., Little J.P. (2021). Elevated Interleukin-10 Levels in COVID-19: Potentiation of Pro-Inflammatory Responses or Impaired Anti-Inflammatory Action?. Front. Immunol..

[19] Voloudakis G., Vicari J.M., Venkatesh S., Hoffman G.E., Dobrindt K., Zhang W., Beckmann N.D., Higgins C.A., Argyriou S., Jiang S. (2022). A translational genomics approach identifies IL10RB as the top candidate gene target for COVID-19 susceptibility. npj Genom. Med..

[20] Singh S., Anshita D., Ravichandiran V. (2021). MCP-1: Function, regulation, and involvement in disease. Int. Immunopharmacol..

[21] Ma A., Zhang L., Ye X., Chen J., Yu J., Zhuang L., Weng C., Petersen F., Wang Z., Yu X. (2021). High Levels of Circulating IL-8 and Soluble IL-2R Are Associated with Prolonged Illness in Patients with Severe COVID-19. Front. Immunol..

[22] Chen Y., Wang J., Liu C., Su L., Zhang D., Fan J., Yang Y., Xiao M., Xie J., Xu Y. (2020). IP-10 and MCP-1 as biomarkers associated with disease severity of COVID-19. Mol. Med..

[23] Saito S., Shahbaz S., Osman M., Redmond D., Bozorgmehr N., Rosychuk R.J., Lam G., Sligl W., Tervaert J.W.C., Elahi S. (2024). Diverse immunological dysregulation, chronic inflammation, and impaired erythropoiesis in long COVID patients with chronic fatigue syndrome. J. Autoimmun..

[24] Khalil B.A., Elemam N.M., Maghazachi A.A. (2021). Chemokines and chemokine receptors during COVID-19 infection. Comput. Struct. Biotechnol. J..

[25] Li L., Dai F., Wang L., Sun Y., Mei L., Ran Y., Ye F. (2023). CCL13 and human diseases. Front. Immunol..

[26] Abbas A.K., Trotta E., Simeonov D.R., Marson A., Bluestone J.A. (2018). Revisiting IL-2: Biology and therapeutic prospects. Sci. Immunol..

[27] Chang Y., Bai M., You Q. (2022). Associations between Serum Interleukins (IL-1*β*, IL-2, IL-4, IL-6, IL-8, and IL-10) and Disease Severity of COVID-19: A Systematic Review and Meta-Analysis. BioMed Res. Int..

[28] Arguinchona L.M., Zagona-Prizio C., Joyce M.E., Chan E.D., Maloney J.P. (2023). Microvascular significance of TGF-β axis activation in COVID-19. Front. Cardiovasc. Med..

[29] Patterson B.K., Guevara-Coto J., Yogendra R., Francisco E.B., Long E., Pise A., Rodrigues H., Parikh P., Mora J., Mora-Rodríguez R.A. (2021). Immune-Based Prediction of COVID-19 Severity and Chronicity Decoded Using Machine Learning. Front. Immunol..

[30] Colarusso C., Maglio A., Terlizzi M., Vitale C., Molino A., Pinto A., Vatrella A., Sorrentino R. (2021). Post-COVID-19 Patients Who Develop Lung Fibrotic-like Changes Have Lower Circulating Levels of IFN-β but Higher Levels of IL-1α and TGF-β. Biomedicines.

[31] Dulfer E.A., Joosten L.A., Netea M.G. (2023). Enduring echoes: Post-infectious long-term changes in innate immunity. Eur. J. Intern. Med..

[32] Rigo S., Barbic F., Khalaf K., Bisoglio A., Pani M., Minonzio M., Rinaldi L., Ciccarelli M., Bordoni M.G., Verzeletti P. (2023). The Long-COVID autonomic syndrome in hospitalized patients: A one-year prospective cohort study. Eur. J. Intern. Med..

[33] Kanno Y. (2023). The uPA/uPAR System Orchestrates the Inflammatory Response, Vascular Homeostasis, and Immune System in Fibrosis Progression. Int. J. Mol. Sci..

[34] Pretorius E., Vlok M., Venter C., Bezuidenhout J.A., Laubscher G.J., Steenkamp J., Kell D.B. (2021). Persistent clotting protein pathology in Long COVID/Post-Acute Sequelae of COVID-19 (PASC) is accompanied by increased levels of antiplasmin. Cardiovasc. Diabetol..

[35] Appelman B., Charlton B.T., Goulding R.P., Kerkhoff T.J., Breedveld E.A., Noort W., Offringa C., Bloemers F.W., van Weeghel M., Schomakers B.V. (2024). Muscle abnormalities worsen after post-exertional malaise in long COVID. Nat. Commun..

